# Risk factors for COVID-19 transmission in England: a multilevel modelling study using routine contact tracing data

**DOI:** 10.1017/S0950268824001043

**Published:** 2024-10-02

**Authors:** Hannah L. Moore, Charlie Turner, Chris Rawlinson, Cong Chen, Neville Q. Verlander, Charlotte Anderson, Gareth J. Hughes

**Affiliations:** 1 UK Field Epidemiology Training Programme, Leeds, UK; 2 Contact Tracing Data Team, UK Health Security Agency, London, UK; 3 The Kids Research Institute Australia, Perth, Australia

**Keywords:** contact tracing, COVID-19, health policy, public health, transmission

## Abstract

Contact tracing for COVID-19 in England operated from May 2020 to February 2022. The clinical, demographic and exposure information collected on cases and their contacts offered a unique opportunity to study secondary transmission. We aimed to quantify the relative impact of host factors and exposure settings on secondary COVID-19 transmission risk using 550,000 sampled transmission links between cases and their contacts. Links, or ‘contact episodes’, were established where a contact subsequently became a case, using an algorithm accounting for incubation period, setting, and contact date. A mixed-effects logistic regression model was used to estimate adjusted odds of transmission. Of sampled episodes, 8.7% resulted in secondary cases. Living with a case (71% episodes) was the most significant risk factor (aOR = 2.6, CI = 1.9–3.6). Other risk factors included unvaccinated status (aOR = 1.2, CI = 1.2–1.3), symptoms, and older age (66–79 years; aOR = 1.4, CI = 1.4–1.5). Whilst global COVID-19 strategies emphasized protection outside the home, including education, travel, and gathering restrictions, this study evidences the relative importance of household transmission. There is a need to reconsider the contribution of household transmission to future control strategies and the requirement for effective infection control within households.

## Introduction

The SARS-CoV-2 virus, which causes COVID-19, is primarily transmitted through droplets from an infected person, infected surfaces and through small, airborne particles (aerosols) [1]. Dynamics of transmission are complex, with major differences in transmission rates associated with where contacts were exposed, household size, symptomatic status and symptom type, vaccination status, socioeconomic status, and national public health policy [2–5]. Spread of the virus mainly occurs between people who are in close contact with each other (less than 1 metre apart) and is associated with poorly ventilated settings, crowded indoor spaces and household contact [6]. Incubation period for the virus can range from around 2–18 days [7] and a person typically becomes infectious around 2 days before symptoms, but this varies according to host susceptibility, vaccination status, and genomic variant [8–10].

After the World Health Organization declared COVID-19 a pandemic in March 2020, guidelines for public health and social measures were issued recommending contact tracing for at least 80% of new cases [11]. Contact tracing is been widely used for preventing and reducing transmission of a number of infectious diseases and data collected from the process can be used to explore epidemiological questions, such as estimating secondary attack rates, routes of transmission, and risk factors for secondary transmission for many diseases [12–14].

The NHS Test and Trace system (NHS T&T) was established in May 2020 in England for contact tracing of COVID-19 cases and their contacts and holds a record of all confirmed cases of COVID-19 (during the period of operation) and their reported contacts, including relevant demographic information and potential exposure events. Routine contact tracing ended in England in February 2022, at which time 15.8 million cases and >31 million contacts had been managed [15].

Although numerous individual studies have sought to understand COVID-19 transmission in specific settings and population groups [16–20], few have quantified transmission in the general population at a national level. The unique nature of the NHS T&T dataset presents an opportunity to accurately assess, with adequate power, the impact of clinical and demographic factors surrounding contact events to inform future public health measures for the control of COVID-19. This is particularly important as understanding the characteristics of cases that are associated with transmission events and the features of the events themselves can help determine which measures are most effective in controlling the spread of COVID-19 and provide evidence for their future use.

## Methods

### Data sources

#### Contact tracing data

Routine contact tracing took place in England from 28 May 2020 in the form of a national-level system (NHS Test and Trace; NHS T&T). NHS T&T holds records of cases (individuals testing positive for SARS-CoV-2 by PCR subsequently referred for contact tracing) and individuals who they may have been in contact with during their infectious period. Information recorded includes details of activities undertaken in the 7 days prior to symptom onset (or positive test for asymptomatic cases), locations visited and other geographical and demographic information. Individuals who were referred to contact tracing on multiple occasions (as a result of re-infection) were recorded as multiple cases within the dataset. Due to updates made to the data collection for location settings on 23 October 2020, only data from 30 October 2020 to 23 February 2022 (end of routine contact tracing) was included in this analysis.

Potential risk factors for transmission (candidate variables) were determined based on data availability and completeness, as well as those documented elsewhere [14]. These included demographic characteristics and clinical information for the case, the type of contextual setting where the contact event took place (such as a workplace setting, shop/supermarket, leisure facility, within the household) and proxy measures for behavioural factors, such as the number of times a contact had been named previously during contact tracing (Table 1). Data on both cases and contacts was captured either by self-completion questionnaire or over the phone by NHS T&T staff. For cases who had previously appeared as a case in the dataset (i.e., the new episode represented a reinfection), individuals were assigned a category to indicate whether they had been a case within 90 days of their sampled case record, more than 90 days ago, or both. These categories align with the testing policy at the time of data collection, which recommended no PCR re-testing within 90 days of a positive result. All cases without a symptom onset date recorded were assumed to be asymptomatic.

**Table 1.** Sample characteristics and secondary attack rate of COVID-19 for sample^a^

		Number of records in sample*	Became a case after contact event (secondary attack rate)	Did not become a case after contact event
		*N*	*N* (percentage)	*N* (percentage)
Total sample		550000	48038 (8.7%)	501962 (91.3%)
Exposing case completed contact tracing		549560		
Yes		544945	47584 (8.7%)	497361 (91.3%)
No		4615	449 (9.7%)	4166 (90.3%)
Sex of the exposing case		547267		
Male		239162	22010 (9.2%)	217152 (90.8%)
Female		288194	25413 (8.8%)	262781 (91.2%)
Prefer not to say or ‘other’		19911	437 (2.2%)	19474 (97.8%)
Setting of the exposure		487887		
Shopping		1144	49 (4.3%)	1095 (95.7%)
Education		13351	369 (2.8%)	12982 (97.2%)
Health care		524	8 (1.5%)	516 (98.5%)
Household		390341	41877 (10.7%)	348464 (89.3%)
Household visitor		28966	1865 (6.4%)	27101 (93.6%)
Leisure/community		15886	829 (5.2%)	15057 (94.8%)
Other activity		3555	155 (4.4%)	3400 (95.6%)
Other workplace		14789	490 (3.3%)	14299 (96.7%)
Personal services		763	18 (2.4%)	745 (97.6%)
Prison/detention facility		79	4 (5.1%)	75 (94.9%)
Social care or home care		613	27 (4.4%)	586 (95.6%)
Travel and commuting		1352	84 (6.2%)	1268 (93.8%)
Visiting friends/relatives		15332	821 (5.4%)	14511 (94.6%)
Working in healthcare		1192	47 (3.9%)	1145 (96.1%)
Age of the exposing case (years)		549432		
0–9		64289	6178 (9.6%)	58111 (90.4%)
10–18		113378	9275 (8.2%)	104103 (91.8%)
19–65		349419	30334 (8.7%)	319085 (91.3%)
66–79		18951	1860 (9.8%)	17091 (90.2%)
80+		3395	379 (11.2%)	3016 (88.8%)
Ethnic group of the exposing case		527961		
White		421495	37595 (8.9%)	383900 (91.1%)
Asian or Asian British		50155	4319 (8.6%)	45836 (91.4%)
Black, African, Caribbean or Black British		14444	1109 (7.7%)	13335 (92.3%)
Mixed or multiple ethnic groups		14694	1293 (8.8%)	13401 (91.2%)
Other ethnic group		7262	585 (8.1%)	6677 (91.9%)
Prefer not to say		19911	437 (2.2%)	19474 (97.8%)
Symptom status of exposing case		550000		
Symptomatic		470947	45240 (9.6%)	425707 (90.4%)
Asymptomatic		79053	2798 (3.5%)	76255 (96.5%)
IMD decile^b^ of residence		545876		
D1 (most deprived)		48655	4101 (8.4%)	44554 (91.6%)
D2		51007	4357 (8.5%)	46650 (91.5%)
D3		52118	4492 (8.6%)	47626 (91.4%)
D4		52757	4642 (8.8%)	48115 (91.2%)
D5		52921	4702 (8.9%)	48219 (91.1%)
D6		55461	4928 (8.9%)	50533 (91.1%)
D7		55181	4848 (8.8%)	50333 (91.2%)
D8		57168	5154 (9.0%)	52014 (91.0%)
D9		58798	5138 (8.7%)	53660 (91.3%)
D10 (least deprived)		61810	5428 (8.8%)	56382 (91.2%)
Vaccination status of the exposing case		521405		
3 doses + 14 days		68616	5404 (7.9%)	63212 (92.1%)
2 doses + 14 days		147899	12481 (8.4%)	135418 (91.6%)
1 dose + 21 days		33978	2723 (8.0%)	31255 (92.0%)
Unvaccinated		270912	25323 (9.3%)	245589 (90.7%)
Number of contacts in the exposure event		549560		
1–3		256187	28051 (10.9%)	228136 (89.1%)
4–9		201293	17391 (8.6%)	183902 (91.4%)
10+		92080	2591 (2.8%)	89489 (97.2%)
Testing pillar of the exposing case		549560		
Pillar 2 (community testing)		500145	46084 (9.2%)	454061 (90.8%)
Not pillar 2		49415	1949 (3.9%)	47466 (96.1%)
Exposed contact completed contact tracing		550000		
Yes		461989	45154 (9.8%)	416835 (90.2%)
No		88011	2884 (3.3%)	85127 (96.7%)
Contact was previously a case		550000		
Not previously		453049	45911 (10.1%)	407138 (89.9%)
Within ≤90 days		70683	782 (1.1%)	69901 (98.9%)
>90 days ago		20000	12 (0.1%)	19988 (99.9%)
Both ≤90 days ago and >90 days ago		6268	1333 (21.3%)	4935 (78.7%)
Number of times exposed contact previously named as a contact in the dataset		550000		
1–3		507905	43884 (8.6%)	464021 (91.4%)
4–9		41806	4129 (8.6%)	37677 (91.4%)
≥10		289	25 (8.7%)	264 (91.3%)
Month/year of exposure episode		550000		
2020	Nov	59327	4804 (8.1%)	54523 (91.9%)
	Dec	35457	4021 (11.3%)	31436 (88.7%)
2021	Jan	20288	1625 (8%)	18663 (92%)
	Feb	90730	8133 (9%)	82597 (91%)
	Mar	30164	2007 (6.7%)	28157 (93.3%)
	Apr	7384	482 (6.5%)	6902 (93.5%)
	May	3849	172 (4.5%)	3677 (95.5%)
	Jun	4044	265 (6.6%)	3779 (93.4%)
	Jul	15916	1345 (8.5%)	14571 (91.5%)
	Aug	36457	2764 (7.6%)	33693 (92.4%)
	Sep	32371	2630 (8.1%)	29741 (91.9%)
	Oct	41848	3140 (7.5%)	38708 (92.5%)
	Nov	50754	4334 (8.5%)	46420 (91.5%)
	Dec	78170	8139 (10.4%)	70031 (89.6%)
2022	Jan	32961	3244 (9.8%)	29717 (90.2%)
	Feb	10280	933 (9.1%)	9347 (90.9%)
Variant of the exposing case		126673		
VOC–20DEC–01		10002	846 (8.5%)	9156 (91.5%)
VOC–20DEC–02		126	2 (1.6%)	124 (98.4%)
VOC–21APR–02		70259	5676 (8.1%)	64583 (91.9%)
VOC–21FEB–02		5	0 (0%)	5 (100%)
VOC–21JAN–02		25	0 (0%)	25 (100%)
VOC–21NOV–01		38888	3577 (9.2%)	35311 (90.8%)
VUI–21APR–01		89	2 (2.2%)	87 (97.8%)
VUI–21APR–03		7	1 (14.3%)	6 (85.7%)
VUI–21FEB–03		89	3 (3.4%)	86 (96.6%)
VUI–21FEB–04		77	1 (1.3%)	76 (98.7%)
VUI–21JAN–01		5	0 (0%)	5 (100%)
VUI–21JUL–01		9	0 (0%)	9 (100%)
VUI–21JUN–01		5	0 (0%)	5 (100%)
VUI–21MAY–01		11	0 (0%)	11 (100%)
VUI–21MAY–02		34	1 (2.9%)	33 (97.1%)
VUI–21OCT–01		4699	429 (9.1%)	4270 (90.9%)
VUI–22JAN–01		2343	183 (7.8%)	2160 (92.2%)
Region of residence for the contact		546692		
East Midlands		46741	4264 (9.1%)	42477 (90.9%)
East of England		65367	6140 (9.4%)	59227 (90.6%)
London		84160	6753 (8.0%)	77407 (92%)
North East		26060	2410 (9.2%)	23650 (90.8%)
North West		71431	6008 (8.4%)	65423 (91.6%)
South East		91983	8141 (8.9%)	83842 (91.1%)
South West		52344	4625 (8.8%)	47719 (91.2%)
West Midlands		57321	5007 (8.7%)	52314 (91.3%)
Yorkshire and Humber		51285	4431 (8.6%)	46854 (91.4%)

* *N* and percentages are not shown for missing data.

a Percentages may not sum due to rounding.

b IMD (Index of Multiple Deprivation) Decile is the official measure of relative deprivation for small areas in England.

The region was allocated according to UKHSA boundaries based on postcode of the contact, which designate seven regions of England with distinct operational responsibilities for health protection [21]. Where contact postcode was not available, postcode of the case was used.

#### Immunization data

The National Immunization Management Service (NIMS) is a database which holds records of COVID-19 vaccinations administered to individuals [14], for public health and health service planning purposes. Contact tracing data was linked to data from NIMS using combinations of NHS number, forename, first initial, surname, date of birth and postcode to obtain vaccination status for cases at the time of each contact event.

#### Index of multiple deprivation

IMD (Index of Multiple Deprivation) Decile is the official measure of relative deprivation for small areas in England. Deprivation decile was assigned according to the postcode of the contact where available, or the exposing case where it was not.

### Definitions

#### Case definition

A case was a laboratory-confirmed case of SARS-CoV-2, as defined in NHS T&T at the time of record. Due to the changing testing requirements in England, this was defined as either: a laboratory PCR (polymerase chain reaction) test, LFD (lateral flow device) test with confirmatory PCR test, or LFD with no negative confirmatory PCR within a specified period.

#### Contact and contact episode definition

A contact was any individual named by a case as being a ‘close contact’ between two days before the date of the case’s onset of symptoms (or test date, if asymptomatic or not reported) and the date of contact tracing. Each contact event that resulted in contact tracing of an individual taking place was termed a ‘contact episode’ and was linked to the specific reported setting where contact took place.

#### Secondary attack rate

The secondary attack rate was defined as the proportion of contacts (as defined above) who became cases themselves within a defined time period.

### Transmission events

Transmission events were detected by linkage of contacts to a subsequent case record within the system where they were notified to the system as a case. Where a link could not be made, it was assumed no transmission had occurred. A simple, deterministic model was applied to define transmission based on operational public health guidelines at the time of contact tracing: the contact episode must have occurred within the likely incubation period for the linked case (between 2 and 14 days before symptom onset or specimen date, inclusive). Where multiple links were identified, a rules-based approach was taken in identifying the most likely transmission link, with each case having a single link. This methodology is further described in a Technical Briefing published by Public Health England [22]. Contact episodes occurring in or among residents of care homes were excluded from the analysis prior to sampling.

### Sampling and power

Given that the NHS T&T dataset contains over 30 million contact episodes, simple random sampling was used to draw a main dataset of 550 000 of these (defined above). This sample size was based on a desired power of 80%, significance level α of 0.05, a minimum odds ratio (OR) of 1.1 and inflated using a design effect of 2 to account for the most complex hypothesized interaction (age category and deprivation decile) and for 25% missing data. A second random sample of 550 000 episodes (excluding those already sampled) was drawn to test model fit, by predicting the probability of transmission using the final model built using the main initial dataset.

### Statistical analysis

As the data was hierarchical in nature, mixed effects (multilevel) logistic regression models were used with random effects of individuals (using a unique identifier applied to all linked records within the dataset) nested within the region of residence, defined *a priori.* Likelihood ratio testing (LRT) was used to obtain *p*-values. Adjusted reference categories were selected to align with those used in existing literature [23] or the largest group, and ‘prefer not to say’ was treated separately to missing data (i.e., included in the model) due to evidence suggesting that those who opt out of answering demographic questions tend to share characteristics and therefore the data should not be considered missing [24, 25].

All analysis was carried out in R version 4.0 with the following packages: tidyverse, lme4, broom.mixed, DHARMa. The BOBYQA [26] optimiser was specified for lme4 mixed-effects models for computational purposes.

#### Single variable analysis

Independent associations with transmission were estimated using a mixed effects (multi-level) logistic regression model. Crude associations with transmission were obtained for each variable by adding that variable separately to a null model containing only the random effects. Estimates were exponentiated to produce an OR (with 95% confidence intervals).

The linearity of continuous variables was assessed using a LRT to measure the cumulative effects of adding the square, cube and fourth power of the variable in three comparisons. A LRT was used to compare model fit at each stage. Where non-linearity was indicated, categorical variables were derived and used in place of the continuous variable to aid interpretation and reduce computation time. Age categories were chosen to align with those used elsewhere [23]. Categories for other variables were derived from visual inspection of the distribution of the data.

#### Multivariable mixed effects modelling

All candidate variables highly significant (*p* < 0.01) in the single variable analysis were considered for inclusion in a multivariable model using a forward-stepwise approach based on hypothesized impact on transmission. Improvement of fit following each variable addition was assessed using a LRT, with variables retained in the model if fit was significantly improved (*p* < 0.05). The Akaike Information Criterion (AIC) was also examined in each step of model-building as an additional relative measure of improvement in model fit for borderline significant variables. The final model was obtained when all candidate variables and interactions had been tested in the model, at which point final *p*-values for categories were calculated using a LRT derived from removing each variable from the final model in turn (*k* − 1). Multicollinearity was checked by comparing the variance inflation factor (VIF) of each independent variable against the dependent variable. Variables with a VIF >10 considered to be highly correlated and the variable with most missing data from a correlated pair was dropped from the model.

Two-way interactions between age/sex variables, age/setting, and symptom/testing route were considered *a priori.* Each interaction was added separately to the model in addition to the main effects after all individual candidate variables had been tested, and retained if their inclusion corresponded to a significant (*p* < 0.05) improvement in fit assessed by LRT.

Variables from the single variable analysis which were considered borderline or non-significant at the 1% level were added to the final main effects model in turn, starting with the most complete, to assess whether they improved model fit.

#### Assessment of fit and model diagnostics

Residuals of the final model were simulated using the DHARMa package [27] for R to aid interpretation and visually examined using quantile-quantile plots. The distribution of outliers was examined using a one-sample Kolomogorov–Smirnov test. Standardized residuals were plotted against predictors and the within-group residual distributions were visually assessed for uniformity. Model fit was examined using the second random sample dataset and predicting probabilities for transmission for this sample using the final model, comparing this to overall secondary attack rate.

The intraclass correlation coefficient (ICC) was calculated for random effects at a value of 1.0, however random effects were retained in the model regardless of results for discussion purposes.

#### Supplementary analyses

Two additional analyses were performed to assess (1) the data quality and relative impact of the addition of an ‘episode variant’ variable and (2) the impact of the interval between symptom onset of the exposing case and the contact event on transmission.

Episode variant was defined as the genetic COVID-19 variant of a case assigned by the testing laboratory following genetic sequencing of the case’s results. The aim of this analysis was to assess whether the time variable served as an adequate proxy for transmissibility for circulating variant, as completeness of the sequencing data made its inclusion unlikely.

The time interval (in days) between symptom onset of the exposing case and the contact event was included in the final model (for symptomatic cases only) to assess its impact on transmission.

## Results

### Study population characteristics

Most of the sampled contact episodes involved a primary case who was symptomatic (*n* = 470 947; 85.6%), tested via Pillar 2 (community) testing (*n* = 500 145; 92.3%) and was of White ethnicity (*n* = 421 495; 79.8%). The primary case was female for 52.6% of sampled contact episodes (*n* = 288 194) and 63.6% of episodes (*n* = 349 419) occurred where the case was aged between 19 and 65 years. For around half of contact episodes, the primary case was unvaccinated (*n* = 270 912; 52.0%).

By month, the highest percentage of recorded episodes occurred in February 2021 (*n* = 90 730; 16.5%), with successive months showing a decrease over spring and early summer 2021, before increasing again in autumn and winter. In the sampled contact episodes, 99.2% cases and 84.0% contacts completed contact tracing.

### Crude secondary attack rates

Overall, 8.7% of contact episodes sampled resulted in the contact latterly becoming a case themselves (Table 1). Among the 550 000 contact episodes sampled, where this information was known, 86.0% of these occurred within a household setting, either as a household member or visitor. The highest secondary attack rate (SAR), 21.3%, was observed among contacts who had been a case at least twice previously (at least once >90 days prior to the contact event and at least once within 90 days of the contact). Higher than average SARs were also observed in household contacts (10.7%), in older age groups (66–79 years, 9.8%; >80 years, 11.2%), those who were contacts of a symptomatic case (9.6%) and those who were contacts during the winter months (range 8.0%–11.3%).

The lowest SAR were observed in April and May 2021 (6.5% and 4.5%), in educational settings (2.8%), healthcare settings (1.5%) and workplaces (3.3%). Personal service (including hairdressers, beauty salons, etc.) settings were also associated with lower-than-average SAR (2.4%). SARs were similar across deprivation decile of residence and ethnic groups.

### Multivariable analysis

#### Model building and diagnostics

Variables described in Table 2 were tested for inclusion in the model as outlined in the methods. Despite initially being non-significant in the single variable analyses, deprivation decile was retained in the model due to significant improvement in model fit. An interaction between symptomatic status and testing pillar also significantly improved fit and was retained in the model.

**Table 2.** Univariable and Multivariable model results for risk factors in COVID-19 transmission

		Crude OR	95% CI	aOR^a^	95% CI	*p*-value	Variance estimate for random effects(SD)
Setting of the exposure							
Shopping (ref)		–	–	–	–	0.000	
Education		0.72	0.53–0.99	0.92	0.66–1.28		
Health care		0.38	0.18–0.81	0.41	0.18–0.93		
Household		2.93	2.17–3.97	2.64	1.92–3.63		
Household visitor		1.61	1.18–2.18	1.50	1.09–2.07		
Leisure/community		1.33	0.97–1.81	1.34	0.97–1.86		
Other activity		1.03	0.73–1.45	1.10	0.77–1.58		
Other workplace		0.86	0.62–1.17	0.84	0.6–1.16		
Personal services		0.34	0.17–0.67	0.28	0.13–0.61		
Prison/detention facility		0.39	0.05–2.86	0.48	0.07–3.6		
Social care or home care		0.78	0.44–1.38	0.93	0.52–1.69		
Travel and commuting		1.55	1.06–2.25	1.61	1.08–2.4		
Visiting friends/relatives		1.37	1.00–1.86	1.36	0.98–1.89		
Working in healthcare		0.75	0.48–1.18	0.83	0.51–1.33		
Age of exposing case							
0–9		0.10	0.09–0.1	0.94	0.9–0.98		
10–18		1.11	1.08–1.15	0.76	0.73–0.79		
19–65 (ref)		–	–	–	–	0.000	
66–79		1.14	1.09–1.2	1.44	1.36–1.52		
80+		1.32	1.19–1.47	1.64	1.45–1.86		
Month/year of exposure episode							
2020	Nov (ref)	–	–	–	–	0.000	
	Dec	1.49	1.4–1.59	1.25	1.17–1.34		
2021	Jan	1.26	1.19–1.35	1.13	1.06–1.22		
	Feb	1.15	1.05–1.25	1.07	0.97–1.18		
	Mar	0.81	0.73–0.9	0.97	0.85–1.09		
	Apr	0.54	0.46–0.64	0.91	0.75–1.11		
	May	0.82	0.72–0.94	1.10	0.94–1.29		
	Jun	1.07	0.99–1.15	1.11	1.01–1.21		
	Jul	0.94	0.88–1	0.92	0.86–0.99		
	Aug	1.01	0.95–1.08	1.11	1.03–1.19		
	Sep	0.93	0.87–0.99	1.22	1.13–1.31		
	Oct	1.00	0.94–1.06	1.27	1.18–1.36		
	Nov	1.07	1.01–1.13	1.35	1.26–1.45		
	Dec	1.34	1.27–1.42	1.53	1.43–1.64		
2022	Jan	1.13	1.06–1.19	1.36	1.27–1.46		
	Feb	0.82	0.76–0.87	1.15	1.06–1.25		
Sex of the exposing case							
Female (ref)		–	–	–	–	0.000	
Male		1.05	1.03–1.07	1.08	1.06–1.11		
Prefer not to say or ‘other’		0.24	0.21–0.26	1.01	0.88–1.16		
IMD decile^b^ of residence of the contact							
D1 (most deprived) (ref)		–	–	–	–	0.000	
D2		1.03	0.98–1.07	1.04	0.99–1.09		
D3		1.04	0.99–1.09	1.06	1.01–1.12		
D4		1.05	1.01–1.10	1.11	1.06–1.16		
D5		1.06	1.02–1.11	1.11	1.06–1.16		
D6		1.06	1.01–1.11	1.11	1.06–1.16		
D7		1.05	1.00–1.09	1.10	1.05–1.16		
D8		1.07	1.03–1.12	1.13	1.08–1.19		
D9		1.04	0.99–1.08	1.09	1.04–1.14		
D10 (least deprived)		1.03	0.99–1.08	1.11	1.05–1.16		
Vaccination status of exposing case							
3 doses + 14 days (ref)		–	–	–	–	0.00	
2 doses + 14 days		1.08	1.04–1.11	1.15	1.11–1.20		
1 dose + 21 days		1.02	0.97–1.07	1.09	1.03–1.15		
Unvaccinated		1.21	1.17–1.25	1.23	1.17–1.29		
Number of contacts in the event							
1–3 (ref)		–	–	–	–	0.00	
4–9		0.77	0.75–0.78	0.85	0.83–0.87		
10+		0.24	0.23–0.25	0.81	0.76–0.86		
Ethnic group of exposing case							
Mixed or multiple ethnic groups		1.00	0.94–1.06	0.98	0.92–1.05		
White (ref)		–	–	–	–	0.000	
Asian or Asian British		0.98	0.95–1.01	0.91	0.88–0.95		
Prefer not to say		0.24	0.21–0.26	0.84	0.78–0.89		
Black, African, Caribbean or Black British		0.87	0.82–0.93	0.82	0.76–0.88		
Other ethnic group		0.92	0.84–1	0.94	0.85–1.03		
Exposed contact completed contact tracing							
Yes		3.19	3.07–3.31	2.28	2.18–2.39		
No (ref)		–	–	–	–	0.000	
Contact was previously a case							
Not previously		–	–	–	–	0.000	
Within ≤90 days		0.10	0.09–0.11	0.07	0.06–0.06		
>90 days ago		0.01	0–0.01	0.00	0–0		
Both ≤90 days ago and >90 days ago		2.39	2.25–2.54	1.99	1.86–2.12		
Number of times exposed contact previously named as a contact in the dataset							
1–3 times (ref)		–		–	–	0.000	
4–9 times		1.00	0.66–1.51	1.21	1.16–1.26		
10+ times		1.15	1.12–1.19	1.29	0.83–1.98		
Symptom status of exposing case							
Symptomatic		2.88	2.77–2.99	–		–	
Asymptomatic (ref)		–	–	–	–	–	
Testing pillar							
Pillar 2 (community testing) (ref)		–	–	–	–	–	
Not Pillar 2 testing		0.41	0.39–0.43	–		–	
Symptom status and testing pillar of case (interaction)							
*Pillar 2 (community) testing*							
Asymptomatic		–	–	–	–	0.00	
Symptomatic		–	–	2.43	2.32–2.54		
*Not Pillar 2 testing*							
Asymptomatic		–	–	0.56	0.48–0.67		
Symptomatic		–	–	1.40	1.18–1.68		
Random effects							
Individual ID							0.000 (0.000)
Region of residence for the contact							0.001 (0.027)

a aOR = adjusted odds ratio. The model has been adjusted for all variables presented in this table.

b IMD (Index of Multiple Deprivation) Decile is the official measure of relative deprivation for small areas in England.

A total of 439 748 contact episodes with complete data were included in the final model (80.0% of the full sample). The model predicted a mean probability of transmission of 9.8% for the first dataset (used for model building) and 9.6% for the second dataset.

Residual plots were approximately normally distributed but included outliers, which were associated with specific months of the year.

#### Risk factors for transmission

After adjustment for clinical and personal characteristics (see Table 2), household exposure was estimated to have by far the strongest association with COVID-19 transmission (aOR = 2.64, 95% CI: 1.92–3.63). Of other exposure settings, being a household visitor (aOR = 1.50, CI: 1.09–2.07) or being a contact during travel or commuting (aOR 1.61, CI: 1.08–2.40) were the only settings significantly associated with increased odds of transmission (Table 2). Settings associated with reduced odds of transmission were personal services (hairdressers, beauty salons, etc.) and contact settings where there were ≥3 other recorded contacts in the exposure setting during the contact event of interest (4–9 contacts: aOR = 0.77, CI: 0.75–0.78; ≥10 contacts: aOR = 0.24, CI: 0.23–0.25).

Different times of the year and individual months were associated with higher odds of transmission, in particular the winter months (Table 2). The month with the largest odds of transmission was December (2020: aOR = 1.49, CI: 1.40–1.59; 2021: aOR = 1.34, CI: 1.27–1.42), followed by January (2021: aOR = 1.26).

Being exposed to cases who were older, male and who were unvaccinated were all factors associated with increased odds of transmission (Table 2). With exposing cases aged 19–65 years as the reference group (loosely working age), being exposed to a case aged ≥66 years was associated with significantly increased odds of transmission (66–79 years: aOR = 1.14, 95% CI: 1.09–1.20; ≥80 years: aOR = 1.32, CI: 1.19–1.47). Being a contact of a male case was weakly associated with a higher odds of transmission (aOR = 1.05, CI: 1.03–1.07) and unvaccinated cases were significantly more likely to transmit to contacts than those fully (3 doses) vaccinated (aOR 1.21, 95% CI: 1.17–1.25). No ethnic group was significantly associated with increased odds of transmission.

Contacts who were previously cases themselves were also more likely to become a case again after a contact event, particularly those who had been cases at least twice previously (aOR = 2.39, CI: 2.25–2.54). There was also a substantial increase in transmission odds for those who completed contact tracing (provided complete details into the system) (aOR = 3.19, CI: 3.07–3.31).

Significant effect modification was observed between the testing pillar and the symptomatic status of the exposing case, with the route for testing for the exposing case modifying the effect of the symptomatic status of the case on transmission to their contacts (Table 2). The odds of transmission from a symptomatic case were 71% higher when the exposing case had been tested through community-based testing compared to all other routes (including hospital-based testing and research related testing; aOR = 2.43 vs. 1.40).

Variance components for random effects, the individual identifier variable and the geographical region variable, were of low magnitude (0.00 and 0.03, respectively) but were retained as levels in the model for discussion purposes.

#### Supplementary analyses

Inclusion of variant in the final mixed effects model did not significantly improve model fit (*p* > 0.05) and examination of VIFs indicated considerable multicollinearity between time and SARS-CoV-2 variant. Data availability for variant was also low (<25% of cases). As the inclusion of month/year as a time variable significantly improved fit of the model (*p* < 0.01), variant was not included in the final model.

The timing of the exposure event relative to symptom onset of the exposing case had substantial impact on the odds of onwards transmission (Supplementary Table 2). This variable was categorized due to its non-linearity and to allow convergence of the model, given the number of variables included. Contact events occurring 6 or more days post-symptom onset were associated with the smallest odds of transmission (aOR = 0.54, CI 0.38–0.77). Contact 1–2 days pre-onset and contact 3–5 days post-onset showed the greatest odds for transmission (aOR range = 1.23–1.24, CI range = 1.16–1.33). Full results tables for the supplementary analyses can be found in the Supplementary Material.

## Discussion

Despite the volume of accumulated COVID-19 research, there remains little evidence of comparative transmission risk across different exposure settings, and even less with a fully adjusted analysis, considering other potential confounding factors. Here, we have exploited the availability of a large, nationally standardized dataset collected for routine contact tracing to obtain robust, independent estimates of COVID-19 transmission risk for different settings in England from October 2020 to February 2022. These estimates are adjusted for temporal changes (including a proxy effect for changing dominant variant), and account for several important individual-level factors. Crucially, linkage to individual vaccination records has enabled estimates of transmission risk to be adjusted for the immunization status of the exposing case.

After adjustment for clinical and personal characteristics, and consistent with other studies [17], household settings were more strongly associated with transmission than any other modifiable factor, both in the context of exposure between residents and from visitors although household secondary attack rate was lower than reported elsewhere [17]. Throughout the pandemic period covered by this analysis, public health policies were informed by modelling studies and largely focused on the prevention of transmission outside households, particularly for large gatherings and where high-risk behaviour was likely [28–30]. Existing studies analyze the spread of disease between household units, with considerable evidence of high-rate transmission occurring at specific events [31–33], however, our analysis indicates that the overall contribution of transmission at these settings was likely much lower compared to the extent at which transmission occurred between household members. Whilst it is true that the public health messaging at the time may have reduced risk for non-household settings relative to households, leading to perceived increased ‘risk’ of transmission, results from a recent study quantifying transmission risk using COVID-19 app-based contact tracing suggests households accounted for 40% of total transmissions, yet only 6% of contacts [34]. These findings have implications for public health policy particularly as the emphasis of non-pharmaceutical interventions globally, largely in the early stages of the pandemic, centred around transmission outside of the home, resulting in border controls, closure of schools and workplaces and many national lockdowns. There may be a place for well-communicated, effective guidance specific to household settings to reduce transmission of COVID-19 and other seasonal respiratory viruses should future large-scale outbreaks arise.

Whilst age and sex of the exposing case impacted transmission risk, possibly due to involvement with personal care or differing contact patterns with others, we did not find ethnicity to be significantly associated. In the UK in particular, ethnic group was considered a risk factor for transmission at various stages of the pandemic, hypothesized to be due to differences in household structures characteristic of some cultures and ethnic groups (larger or multi-generational households) [35]. A similar finding was not demonstrated here, however, it was not possible to include indicators of household size, property type or occupation of household members. The lack of variance explained at an individual-level may demonstrate the importance of contact event characteristics over individual characteristics, for example, a greater risk of transmission due to closer or more sustained contact within some settings, rather than an intrinsically greater risk of transmission from individual age groups or ethnicities.

Additionally, the increased risk of transmission observed in unvaccinated cases reinforces the role of individual health factors in outbreak control policies. Symptomatic cases were significantly associated with increased transmission to their contacts, which supports the abundance of existing literature in this area [8, 36, 37]. We began to explore this further in our supplementary analysis demonstrating a specific window of time from symptom onset to contact associated with higher transmission risk, building on other work which has used contact tracing data linked to genomic sequencing data to explore the role of specific COVID-19 variants and viral load in transmission [10]. Further work is still needed to explore this in relation to newer and more established COVID-19 genetic variants and their respective viral loads and incubation periods. The interaction between symptomatic status and testing pillar is expected, given pillar 2 testing criteria in England required a case to be symptomatic. Being a contact previously appearing as a case within the dataset, specifically as a case more than 90 days and 90 or less days ago from the contact event, was associated with higher odds of transmission, potentially due to the role of this variable as a proxy indicator for high levels of social mixing or poor immune response to the virus.

### Limitations and biases

Data on many factors known to be important for SARS-CoV-2 transmission were not captured during routine contact tracing (such as household size, precise proximity, and duration of exposure). While proxy variables were used where possible, the impact of this is uncertain. The genomic variant of SARS-CoV-2 from the exposing case could not be modelled due to data quality issues, however, inclusion of month-time was an adequate proxy for the comparative transmissibility of variants.

The nature and structure of the dataset introduces bias; notably, those who provided more complete details into the system were more likely to be successfully linked to their own records and transmission identified. Similarly, settings where individuals are familiar to their contacts (households, workplaces) are likely to result in more complete contact tracing information and successful linkage. The deterministic detection of transmission events also prioritized contact occurring in households, meaning if there were multiple plausible contact events for a case, events occurring within a household were preferentially attributed to transmission. However, the determination rules were created based on biological plausibility and likelihood and the number of times a contact had previously been named in the dataset was adjusted for in the analysis.

## Conclusion

During the COVID-19 pandemic in England between May 2020 and February 2022, household exposure was the most important risk factor for COVID-19 transmission. Symptomatic status and vaccination status were also highly important. The prominence of the household setting in COVID-19 transmission highlights a clear need for pragmatic, well-communicated guidance on effective ways to reduce transmission within the home, however, the relative insignificance of other exposure settings in the role of transmission is more difficult to interpret. Linking large, routine datasets to generate empirical evidence for disease transmission can provide valuable insights into infectious disease epidemiology which can be used to inform policy and control programmes. Factoring this into the design of such datasets could provide powerful potential to address evidence gaps where very large sample sizes are required.

## Supporting information

Moore et al. supplementary material Moore et al. supplementary material

## Data Availability

Analyses were limited to the secondary use of datasets routinely collected as part of the public health function of the UKHSA under regulation 3 of the United Kingdom 2010 health services act and therefore ethical approval was not deemed necessary. For the same reason, data cannot be made publicly available. Applications for requests to access relevant anonymised data included in this study should be submitted to the UKHSA Office for Data Release (https://www.gov.uk/government/publications/accessing-ukhsa-protected-data/accessing-ukhsa-protected-data) and may be subject to legal restrictions.

## Supplementary material

For supplementary material accompanying this paper visit http://doi.org/10.1017/S0950268824001043. click here to view supplementary material
