# Supplementary material for: Risk factors for COVID-19 transmission in England: a multilevel modelling study using routine contact tracing data
Source: Epidemiol Infect. 2024 Oct 2;152:e112. doi: 10.1017/S0950268824001043 (PMC11450508; doi:10.1017/S0950268824001043)
Supplement: Moore et al. supplementary material [file S0950268824001043sup001.docx]

Epidemiology and Infection

**Risk factors for COVID-19 transmission in England: a multilevel modelling study using routine contact tracing data**

Moore H L, Turner C, Rawlinson C, Chen C, Verlander N Q, Anderson C *, Hughes G J*

*Authors contributed equally to this work

**Supplementary material**

Contents:

Table S1 – Characteristics of data included in final model

Table S2 – Table S2 – Supplementary analysis: Multivariable model results for risk of increased time from symptom onset to contact event

## Table S1 – Characteristics of data included in final model^+^

|  | | Number of records retained in model | Became a case after contact event (secondary attack rate) | Did not become a case after contact event |
| --- | --- | --- | --- | --- |
| **Total sample** | | **439748** | 43051 (9.8%) | 396697 (90.2%) |
| **Sex of the exposing case** | | **439748** |  |  |
| Male | | 193072 | 19680 (10.2%) | 173392 (89.8%) |
| Female (ref) | | 242602 | 23095 (9.5%) | 219507 (90.5%) |
| Perfer not to say or 'other' | | 4074 | 276 (6.8%) | 4074 (93.2%) |
| **Setting of the exposure** | | **439748** |  |  |
| Shopping (ref) | | 1011 | 40 (4.0%) | 971 (96%) |
| Education | | 12373 | 363 (2.9%) | 12010 (97.1%) |
| Health care | | 477 | 7 (1.5%) | 470 (98.5%) |
| Household | | 350173 | 38737 (11.1%) | 311436 (88.9%) |
| Household visitor | | 26672 | 1651 (6.2%) | 25021 (93.8%) |
| Leisure/community | | 14901 | 778 (5.2%) | 14123 (94.8%) |
| Other activity | | 3260 | 138 (4.2%) | 3122 (95.8%) |
| Other workplace | | 13208 | 447 (3.4%) | 12761 (96.6%) |
| Personal services | | 678 | 8 (1.2%) | 670 (98.8%) |
| Prison / detention facility | | 58 | 1 (1.7%) | 57 (98.3%) |
| Social care or home care | | 494 | 16 (3.2%) | 478 (96.8%) |
| Travel and commuting | | 1220 | 71 (5.8%) | 1149 (94.2%) |
| Visiting friends/relatives | | 14158 | 762 (5.4%) | 13396 (94.6%) |
| Working in healthcare | | 1065 | 32 (3.0%) | 1033 (97%) |
| **Age of the exposing case** | | **439748** |  |  |
| 0-9 | | 56131 | 5696 (10.1%) | 50435 (89.9%) |
| 10-18 | | 98925 | 8463 (8.6%) | 90462 (91.4%) |
| 19-65 (ref) | | 268672 | 26919 (10%) | 241753 (90%) |
| 66-79 | | 13404 | 1652 (12.3%) | 11752 (87.7%) |
| 80+ | | 2616 | 321 (12.3%) | 2295 (87.7%) |
| **Ethnic group of the exposing case** | | **439748** |  |  |
| Mixed or multiple ethnic groups | | 12415 | 1194 (9.6%) | 11221 (90.4%) |
| White (ref) | | 358025 | 35384 (9.9%) | 322641 (90.1%) |
| Asian or Asian British | | 40617 | 3905 (9.6%) | 36712 (90.4%) |
| Prefer not to say | | 12538 | 1120 (8.9%) | 11418 (91.1%) |
| Black African Caribbean or Black British | | 11037 | 949 (8.6%) | 10088 (91.4%) |
| Other ethnic group | | 5116 | 499 (9.8%) | 4617 (90.2%) |
| **Symptomatic status of exposing case** | | **439748** |  |  |
| Symptomatic | | 388658 | 40727 (10.5%) | 347931 (89.5%) |
| Asymptomatic (ref) | | 51090 | 2324 (4.5%) | 48766 (95.5%) |
| **Vaccination status of exposing case** | | **439748** |  | |
| 3 doses + 14 days (ref) | | 57335 | 5161 (9%) | 52174 (91%) |
| 2 doses + 14 days | | 115865 | 11895 (10.3%) | 103970 (89.7%) |
| 1 dose + 21 days | | 30284 | 2580 (8.5%) | 27704 (91.5%) |
| Unvaccinated | | 236264 | 23415 (9.9%) | 212849 (90.1%) |
| **Number of contacts in the exposure event** | | **439748** |  | |
| 1-3 (ref) | | 230216 | 25557 (11.1%) | 204659 (88.9%) |
| 4-9 | | 180900 | 15849 (8.8%) | 165051 (91.2%) |
| 10+ | | 28632 | 1645 (5.7%) | 26987 (94.3%) |
| **Testing pillar of exposing case** | | **439748** |  | |
| Pillar 2 (community testing) | | 421576 | 41674 (9.9%) | 379902 (90.1%) |
| Not pillar 2 | | 18172 | 1377 (7.6%) | 16795 (92.4%) |
| **Contact completed contact tracing** | | **439748** |  | |
| Yes | | 376722 | 40614 (10.8%) | 336108 (89.2%) |
| No (ref) | | 63026 | 2437 (3.9%) | 60589 (96.1%) |
| **Contact was previously a case** | | **439748** |  | |
| Not previously known case (ref) | | 357046 | 41144 (11.5%) | 315902 (88.5%) |
| Within ≤90 days | | 62510 | 684 (1.1%) | 61826 (98.9%) |
| >90 days ago | | 14881 | 9 (0.1%) | 14872 (99.9%) |
| Both ≤90 days ago and >90 days ago | | 5311 | 1214 (22.9%) | 4097 (77.1%) |
| **Number of times exposed contact named as a contact** | | **439748** |  |  |
| 1-3 times (ref) | | 403276 | 39316 (9.7%) | 363960 (90.3%) |
| 4-9 times | | 36211 | 3710 (10.2%) | 32501 (89.8%) |
| 10+ times | | 261 | 25 (9.6%) | 236 (90.4%) |
| **Month/year** | | **439748** |  |  |
| 2020 | Nov (ref) | 16709 | 1363 (8.2%) | 15346 (91.8%) |
|  | Dec | 29415 | 3422 (11.6%) | 25993 (88.4%) |
| 2021 | Jan | 25219 | 2642 (10.5%) | 22577 (89.5%) |
|  | Feb | 7540 | 729 (9.7%) | 6811 (90.3%) |
|  | Mar | 4198 | 356 (8.5%) | 3842 (91.5%) |
|  | Apr | 1678 | 124 (7.4%) | 1554 (92.6%) |
|  | May | 2261 | 200 (8.8%) | 2061 (91.2%) |
|  | Jun | 11156 | 1033 (9.3%) | 10123 (90.7%) |
|  | Jul | 26806 | 2182 (8.1%) | 24624 (91.9%) |
|  | Aug | 26334 | 2440 (9.3%) | 23894 (90.7%) |
|  | Sep | 30882 | 2928 (9.5%) | 27954 (90.5%) |
|  | Oct | 46580 | 4504 (9.7%) | 42076 (90.3%) |
|  | Nov | 39529 | 4063 (10.3%) | 35466 (89.7%) |
|  | Dec | 69228 | 7628 (11%) | 61600 (89%) |
| 2022 | Jan | 78570 | 7581 (9.6%) | 70989 (90.4%) |
|  | Feb | 23643 | 1856 (7.9%) | 21787 (92.1%) |
| **IMD decile^§^ of residence of the contact** | | **439748** |  |  |
| D1 (most deprived) (ref) | | 39249 | 3605 (9.2%) | 35644 (90.8%) |
| D2 | | 40472 | 3844 (9.5%) | 36628 (90.5%) |
| D3 | | 41017 | 3956 (9.6%) | 37061 (90.4%) |
| D4 | | 41670 | 4161 (10%) | 37509 (90%) |
| D5 | | 42565 | 4253 (10%) | 38312 (90%) |
| D6 | | 44645 | 4437 (9.9%) | 40208 (90.1%) |
| D7 | | 44595 | 4423 (9.9%) | 40172 (90.1%) |
| D8 | | 46608 | 4688 (10.1%) | 41920 (89.9%) |
| D9 | | 48068 | 4703 (9.8%) | 43365 (90.2%) |
| D10 (least deprived) | | 50859 | 4981 (9.8%) | 45878 (90.2%) |
| **Region of residence for the contact** | | **439748** |  |  |
| East Midlands | | 39032 | 3911 (10%) | 35121 (90%) |
| East of England | | 53809 | 5609 (10.4%) | 48200 (89.6%) |
| London | | 58325 | 5772 (9.9%) | 52553 (90.1%) |
| North East | | 21498 | 2175 (10.1%) | 19323 (89.9%) |
| North West | | 56684 | 5347 (9.4%) | 51337 (90.6%) |
| South East | | 76023 | 7445 (9.8%) | 68578 (90.2%) |
| South West | | 44836 | 4274 (9.5%) | 40562 (90.5%) |
| West Midlands | | 47582 | 4506 (9.5%) | 43076 (90.5%) |
| Yorkshire and Humber | | 41959 | 4012 (9.6%) | 37947 (90.4%) |

+ Percentages may not sum due to rounding

§ IMD (Index of Multiple Deprivation) Decile is the official measure of relative deprivation for small areas in England.

## Table S2 – Supplementary analysis: Multivariable model results for risk of increased time from symptom onset to contact event

|  | | aOR* | 95% CI | p-value  (LR test compared to model with k-1) | Variance estimate for random effects (SD) |
| --- | --- | --- | --- | --- | --- |
| **Setting of the exposure** | |  |  |  |  |
| Shopping (ref) | | - | - | 0.00 |  |
| Education | | 0.99 | 0.7 - 1.41 |  |  |
| Health care | | 0.39 | 0.16 - 0.93 |  |  |
| Household | | 3.25 | 2.33 - 4.54 |  |  |
| Household visitor | | 1.61 | 1.15 - 2.25 |  |  |
| Leisure/community | | 1.41 | 1.00 - 1.98 |  |  |
| Other activity | | 1.15 | 0.79 - 1.67 |  |  |
| Other workplace | | 0.89 | 0.63 - 1.26 |  |  |
| Personal services | | 0.31 | 0.14 - 0.66 |  |  |
| Prison / detention facility | | 0.54 | 0.07 - 4.00 |  |  |
| Social care or home care | | 0.99 | 0.54 - 1.83 |  |  |
| Travel and commuting | | 1.76 | 1.17 - 2.66 |  |  |
| Visiting friends/relatives | | 1.43 | 1.02 - 2.01 |  |  |
| Working in healthcare | | 0.78 | 0.47 - 1.29 |  |  |
| **Age of the exposing case** | |  |  |  |  |
| 0-9 | | 0.92 | 0.88 - 0.96 |  |  |
| 10-18 | | 0.76 | 0.73 - 0.79 |  |  |
| 19-65 (ref) | | - | - | 0.00 |  |
| 66-79 | | 1.45 | 1.37 - 1.53 |  |  |
| 80+ | | 1.64 | 1.44 - 1.87 |  |  |
| **Month/year of exposure episode** | |  |  |  |  |
| 2020 | Nov(ref) | - | - | 0.00 |  |
|  | Dec | 1.24 | 1.16 - 1.33 |  |  |
| 2021 | Jan | 1.11 | 1.03 - 1.19 |  |  |
|  | Feb | 1.06 | 0.96 - 1.17 |  |  |
|  | Mar | 0.93 | 0.82 - 1.06 |  |  |
|  | Apr | 0.86 | 0.70 - 1.06 |  |  |
|  | May | 1.07 | 0.91 - 1.26 |  |  |
|  | Jun | 1.07 | 0.98 - 1.17 |  |  |
|  | Jul | 0.90 | 0.83 - 0.97 |  |  |
|  | Aug | 1.07 | 1.00 - 1.16 |  |  |
|  | Sep | 1.18 | 1.09 - 1.27 |  |  |
|  | Oct | 1.22 | 1.13 - 1.31 |  |  |
|  | Nov | 1.31 | 1.22 - 1.41 |  |  |
|  | Dec | 1.47 | 1.37 - 1.57 |  |  |
| 2022 | Jan | 1.29 | 1.20 - 1.38 |  |  |
|  | Feb | 1.11 | 1.02 - 1.20 |  |  |
| **Sex of the exposing case** | |  |  |  |  |
| Female (ref) | | - | - | 0.00 |  |
| Male | | 1.08 | 1.06 - 1.11 |  |  |
| Prefer not to say or 'other' | | 1.08 | 0.93 - 1.25 |  |  |
| **IMD decile^§^ of residence of the contact** | |  |  |  |  |
| D1 (most deprived) (ref) | | - | - |  |  |
| D2 | | 1.03 | 0.98 - 1.08 |  |  |
| D3 | | 1.06 | 1.00 - 1.11 |  |  |
| D4 | | 1.10 | 1.05 - 1.16 |  |  |
| D5 | | 1.10 | 1.05 - 1.16 |  |  |
| D6 | | 1.10 | 1.05 - 1.16 |  |  |
| D7 | | 1.10 | 1.04 - 1.15 |  |  |
| D8 | | 1.13 | 1.08 - 1.19 |  |  |
| D9 | | 1.07 | 1.02 - 1.13 |  |  |
| D10 (least deprived) | | 1.10 | 1.04 - 1.15 |  |  |
| **Vaccination status of exposing case** | |  |  |  |  |
| 3 doses + 14 days (ref) | | - | - | 0.00 |  |
| 2 doses + 14 days | | 1.14 | 1.1 - 1.19 |  |  |
| 1 dose + 21 days | | 1.07 | 1.01 - 1.14 |  |  |
| Unvaccinated | | 1.20 | 1.15 - 1.27 |  |  |
| **Number of contacts in the exposure event** | |  |  |  |  |
| 1-3 (ref) | | - | - | 0.00 |  |
| 4-9 | | 0.85 | 0.83 - 0.87 |  |  |
| 10+ | | 0.80 | 0.75 - 0.85 |  |  |
| **Ethnic group of exposing case** | |  |  |  |  |
| Mixed or multiple ethnic groups | | 0.99 | 0.93 - 1.06 |  |  |
| White (ref) | | - | - | 0.00 |  |
| Asian or Asian British | | 0.92 | 0.88 - 0.95 |  |  |
| Prefer not to say | | 0.85 | 0.8 - 0.91 |  |  |
| Black, African, Caribbean or Black British | | 0.83 | 0.77 - 0.9 |  |  |
| Other ethnic group | | 0.97 | 0.88 - 1.07 |  |  |
| **Time from symptom onset of case to contact event** | |  |  |  |  |
| Contact on day of symptom onset (ref) | | - | - | 0.00 |  |
| Contact 6+ days pre-onset | | 1.69 | 0.85 - 3.36 |  |  |
| Contact 3-5 days pre-onset | | 1.19 | 0.82 - 1.74 |  |  |
| Contact 1-2 days pre-onset | | 1.24 | 1.16 - 1.32 |  |  |
| Contact 1-2 days post-onset | | 1.22 | 1.16 - 1.28 |  |  |
| Contact 3-5 days post-onset | | 1.23 | 1.14 - 1.33 |  |  |
| Contact 6+ days post-onset | | 0.54 | 0.38 - 0.77 |  |  |
| **Contact completed contact tracing** | |  |  |  |  |
| Yes | | 2.27 | 2.17 - 2.38 |  |  |
| No | | - | - | 0.00 |  |
| **Contact was previously a case** | |  |  |  |  |
| Not previously (ref) | | - | - | 0.00 |  |
| Within ≤90 days | | 0.07 | 0.06 - 0.07 |  |  |
| >90 days ago | | 0.00 | 0 - 0.01 |  |  |
| Both ≤90 days ago and >90 days ago | | 2.00 | 1.86 - 2.14 |  |  |
| **Times exposed contact named in dataset** | |  |  |  |  |
| 1-3 times (ref) | | - | - | 0.00 |  |
| 4-9 times | | 1.20 | 1.15 - 1.25 |  |  |
| 10+ times | | 1.33 | 0.86 - 2.06 |  |  |
| **Testing pillar of exposing case** | |  |  |  |  |
| Pillar 2 (community testing) (ref) | | - | - | 0.000 |  |
| Not Pillar 2 testing | | 0.77 | 0.72 - 0.83 |  |  |
| **Random effects** | |  |  |  |  |
| Individual ID | | - | - |  | 0.000 (0.000) |
| Region of residence for the contact | | - | - |  | 0.000 (0.026) |

*aOR = adjusted odds ratio. The model has been adjusted for all variables shown in the table.
§ IMD (Index of Multiple Deprivation) Decile is the official measure of relative deprivation for small areas in England.
